# Progress in Surgery of the Peritoneum

**Published:** 1901-01-26

**Authors:** 


### PROGRESS IN SURGERY OF THE PERITONEUM.

**The Power of Absorption** of the peritoneum is important from an operative point of view. Robinson[1] formulates the following conclusions on this point:—1. The primary path by which fluids pass from the peritoneum into the circulation is by the way of the lymphatics. The secondary path is the blood vessels. 2. A stream of fluid exists in the peritoneum directed towards the diaphragm. 3. The anatomical structure, physiological function (respiration), of the diaphragm enables it to act like a suction or force pump. 4. The diaphragm is the primary locality of peritoneal absorption of solid granules. 5. The vast function of absorption performed by the diaphragm and the very small part taken in this process by other portions of the peritoneum account for the non-fatality in cases in which there is a purulent condition found at the oviductal ends and about the appendix, and when there is a ruptured gall bladder, which is circumscribed and confined by the ligamentum hepatico-colicum. The virulent microbes or their products are not absorbed but circumscribed. 6. On account of vigorous absorptive powers, the nearer peritonitis approaches the diaphragm the more dangerous it is to life. 7. When foreign bodies (microbes or coloured granules) enter the peritoneum the leucocytes swarm out (*a*) to digest the invader; (*b*) to surround or imprison the microbe; or (*c*) to sterilise the germ. 8. The normal peritoneum is automatic in regulating the quantity of fluids contained. Normally it will absorb all excessive fluid; but in abnormal conditions it may only add to the fluid injected. 9. It appears to be the leucocyte which carries the coloured granules from the peritoneal cavity through the stomata vera into the sub-peritoneal diaphragmatic lymphatics. 10. The diaphragm absorbs fluids perhaps by imbibition. Imbibition is molecular when a mass of tissue absorbs the fluid, and capillary when it passes through the pores of the vessels.

**Peritonitis.**—Berg[2] summarises the views of different observers on the question of peritonitis as follows: 1. Bacteria alone, except when in very large quantity, are not sufficient to produce an exudative peritonitis. 2. This immunity on the part of the healthy peritoneum to the deleterious action of bacteria is due to its very rapid resorptive power. 3. The resorption under normal conditions is mainly by way of the blood vessels. 4. Peritoneal resorption is an active cellular process, and not a passive one. This cellular activity is manifested in the diseased as well as in the healthy condition, but to a somewhat lessened degree. The more marked the disease the greater is the interference with the activity, it being least when the peritoneal endothelium has been considerably altered, and ceasing altogether with the destruction of these cells, resorption by much modified peritoneum being similar to resorption by other types of cellular tissue. As bearing out these points he maintains that the treatment of exudative peritonitis which has yielded him the best results is cœliotomy, the site of the incision depending on the cause of the peritonitis; removal of the acting cause; if the intestine is very much distended, incision of one of the most distended coils and evacuation of its contents; suture of the bowel if its muscular wall is not paralysed, otherwise drainage of the proximal end of the bowel; removal of the exudate from the free peritoneal cavity by moist sponges; protection of all raw spaces or modified peritoneal surfaces by gauze; closure of the rest of the abdominal wound; in addition to the general systemic treatment for the toxæmia and bacteræmia. The percentage of recoveries diminishes considerably as the duration of the disease increases. Dowd[3] gives some cases illustrating the ability of the peritoneum to overcome sepsis, and Wallace[4] mentions a case of suppurative peritonitis which recovered after spontaneous evacuation.

**Shock and Infection in Abdominal Operations** are considered by Turck,[5] who forms the following conclusions. 1. The skin is a source of infection that may cause death, though all usual precautions are particularly taken. Susceptibility to infection is produced in cases of lowered vitality and weakened condition; or in cases in which shock is present from whatever cause, in a mild or severe form. 2. The present methods yet in use of towels and laparotomy sheets, do not sufficiently protect the field of operation. 3. The use of a rubber protector which is made to fit close to the skin more thoroughly prevents the danger of infection from the skin, and also lessens the liability of the skin becoming contaminated from the escape of visceral contents, pus, or other infected material occurring during the operation. 4. When death occurs from supposed "shock" or "exhaustion," especially when the viscera have been exposed to dangers of infection by the escape of visceral contents, the death is probably due more to the infection than to "shock" or "exhaustion." 5. Whenever practicable the viscera may be drawn through the small openings of a protective rubber shield overlapping the abdominal wound, and absolute protection of the abdominal cavity is secured. 6. Exposed viscera may be covered with a rubber hood, kept warm by small rubber hot-water bags, thus preventing contamination as well as lessening susceptibility to infection. 7. Animals naturally immune to certain bacteria are rendered susceptible by exposure of the viscera to the air for an hour or more. 8. Subcutaneous or intravenous infusion of physiological salt solution does not materially lessen the susceptibility of an animal, after the exposure and manipulation of the viscera. 9. When heat at 48° or 50° C. is applied within the abdominal cavity during the time corresponding to the exposure and manipulation of the viscera inoculation by pathogenic or non-pathogenic germs seldom results in infection and death. 10. Susceptibility to infection is not materially decreased by the external application of heat.

11. In severe operations, when extreme shock or collapse is present, resuscitation is best accomplished by the application of heat within the stomach and abdominal cavity.

**Subdiaphragmatic Abscess.**—Godlee[6] classifies the causes of this condition under the following heads: 1. Stomach.—If a gastric ulcer ruptured the pus might be localised, even though it had been poured out into the peritoneal cavity. In other cases the rupture might occur into the peritoneal cavity. These chronic abscesses burrow greatly, and when accompanied by serous effusion into the pleura are likely to be mistaken for cases of empyema. 2. Intestine.—In these cases the abscess results from the rupture of a duodenal ulcer, or an ulcer of the transverse colon. There might be no dulness when the patient is lying down, but a tympanitic note; yet if the patient stands erect some dulness may be evident at the lower part. Drainage may be sufficient in these cases, but it is sometimes possible to close the opening in the bowel. 3. Cæcum and Appendix.—When a subdiaphragmatic abscess follows on disease of the cæcum or appendix, the original inguinal swelling might become less obvious. 4. Hydatids of the liver or spleen. 5. Abscess of the liver and bile passages. 6. Perinephric abscess and pyonephrosis. 7. Subcutaneous Injuries.—The abscess sometimes follows a blow without a wound on the skin: such a blow probably causes a rupture of a previously existing ulcer. 8. Wounds may give rise to the abscess by the introduction of septic material. 9. Metastases. 10. Caries or necrosis of the ribs. 11. Collections of pus in the thorax might make their way downwards. 12. Various Causes.—Actinomycosis, tuberculosis, peritonitis, and disease of the pancreas.

[1] Med. Rec. July 28, 1900, p. 126. [2] Ibid. June 30, 1900, p. 1115. [3] Ann. of Surg. July, 1900, p. 139. [4] Lancet, July 7, 1900, p. 17. [5] Med. Rec., Aug. 11, 1900, p. 216. [6] Lancet, Aug. 4, 1900, p. 334.

